# Complete chloroplast genome sequences of five *Bruguiera* species (Rhizophoraceae): comparative analysis and phylogenetic relationships

**DOI:** 10.7717/peerj.12268

**Published:** 2021-10-22

**Authors:** Panthita Ruang-areerate, Wasitthee Kongkachana, Chaiwat Naktang, Chutima Sonthirod, Nattapol Narong, Nukoon Jomchai, Pasin Maprasop, Chatree Maknual, Nawin Phormsin, Jeremy R. Shearman, Wirulda Pootakham, Sithichoke Tangphatsornruang

**Affiliations:** 1 National Omics Center, National Science and Technology Development Agency (NSTDA), Pathum Thani, Thailand; 2 Department of Marine and Coastal Resources, Bangkok, Thailand

**Keywords:** Bruguiera, Mangrove, Chloroplast genome, Rhizophoraceae, Comparative analysis, Phylogenetic relationships

## Abstract

*Bruguiera* is a genus of true mangroves that are mostly distributed in the Indo-West Pacific region. However, the number of published whole chloroplast genome sequences of *Bruguiera* species are limited. Here, the complete chloroplast sequences of five *Bruguiera* species were sequenced and assembled using Illumina data. The chloroplast genomes of *B. gymnorhiza*, *B. hainesii, B. cylindrica*, *B. parviflora* and *B. sexangula* were assembled into 161,195, 164,295, 164,297, 163,228 and 164,170 bp, respectively. All chloroplast genomes contain 37 tRNA and eight rRNA genes, with either 84 or 85 protein-coding genes. A comparative analysis of these genomes revealed high similarity in gene structure, gene order and boundary position of the LSC, SSC and two IR regions. Interestingly, *B. gymnorhiza* lost a *rpl32* gene in the SSC region. In addition, a *ndhF* gene in *B. parviflora* straddles both the SSC and IRB boundary regions. These genes reveal differences in chloroplast evolution among *Bruguiera* species. Repeats and SSRs in the chloroplast genome sequences were found to be highly conserved between *B. cylindrica* and *B. hainesii* as well as *B. gymnorhiza* and *B. sexangula* indicating close genetic relationships based on maternal inheritance. Notably, *B. hainesii*, which is considered a hybrid between *B. gymnorhiza* and *B. cylindrica*, appears to have inherited the chloroplast from *B. cylindrica*. Investigating the effects of selection events on shared protein-coding genes showed a positive selection in *rps7* and *rpl36* genes in all species compared to land-plant species. A phylogenetic analysis, based on 59 conserved chloroplast protein-coding genes, showed strong support that all *Bruguiera* species are in the clade Rhizophoraceae. This study provides valuable genetic information for the study of evolutionary relationships and population genetics in *Bruguiera* and other mangrove species.

## Introduction

Mangroves are plants that live in coastal regions across tropical and sub-tropical regions. They play an important role in ecology by providing habitats and nurseries for many marine organisms and protecting coastlines from erosion. There are approximately 70 mangrove species belonging to 16 families (Kathiresan & Bingham, 2001). Rhizophoraceae is a true mangrove family consisting of ~18 species in four genera, which are *Bruguiera*, *Ceriops*, *Kandelia* and *Rhizophora* (Tomlinson, Primack & Bunt, 1979).

*Bruguiera* is a dominant and economically important mangrove genus. It produces wood that is used for fuel and house construction, and produces several useful metabolites such as diterpenes and triterpenes (Dahdouh-Guebas et al., 2000; Han et al., 2004; Nebula, Harisankar & Chandramohanakumar, 2013). This genus comprises six species, namely *B. cylindrica*, *B. exaristata*, *B. gymnorhiza*, *B. hainesii*, *B. parviflora* and *B. sexangula* as well as their hybrids such as *B. x rhynchopetala* and *B. x dungarra* (Duke & Ge, 2011; Duke & Kudo, 2018). Among them, *B. gymnorhiza* is the most widespread mangrove plant, while *B. hainesii* is a critically endangered mangrove plant (FAO, 2007; Duke & Ge, 2011). Most *Bruguiera* species are distributed in the Indo-West Pacific region, except *B. exaristata* that is mostly distributed in Northern Australia and Southern New Guinea (FAO, 2007; Duke & Ge, 2011; Urashi et al., 2013). Based on morphological characters, the genus *Bruguiera* is separated into two major groups (Hou, 1958; Sheue, Yong & Yang, 2005; Duke & Ge, 2011). One group (*B. gymnorhiza, B. exaristata* and *B. sexangula*) has large leaves and large solitary-flowered inflorescences, while the other group (*B. cylindrica*, *B. hainesii and B. parviflora*) has small leaves, small multiple-flowered inflorescences. In mangrove forests, the distributions of *Bruguiera* species overlap each other and it can be difficult to identify species based on morphological characters (Abeysinghe et al., 1999; Duke & Ge, 2011). Consequently, molecular markers such as random amplified polymorphic DNA (RAPD) and simple sequence repeats (SSRs) based on nuclear and chloroplast sequences have been developed for species identification in *Bruguiera* (Abeysinghe et al., 1999; Islam et al., 2008).

Chloroplasts are semi-autonomous organelles in algae, cyanobacteria and plants that are responsible for photosynthesis. Chloroplast genomes have been used to study plant species identification, population genetics, genome evolution and phylogenetics (Shi et al., 2005; Dong et al., 2012; Saddhe, Jamdade & Kumar, 2016; Bi et al., 2018; Wu et al., 2019). For example, DNA barcoding based on chloroplast markers such as *matK*, *rbcL* and some intergenic spacer regions (*i.e*., *trnL*-*trnF* and *trnH*-*psbA*) is an effective tool to identify mangrove species and their evolutionary relationships (Shi et al., 2005; Saddhe, Jamdade & Kumar, 2016; Wu et al., 2019). In most land plants, chloroplast genomes are inherited maternally. The chloroplast structure is usually a small circular quadripartite DNA consisting of one large single copy (LSC) region, one small single copy (SSC) region and two inverted repeats (IRs), which separate the LSC and SSC (Mower & Vickrey, 2018). Some plant lineages have lost one copy of the IR, such as papilionoid legumes (Palmer & Stein, 1986; Choi, Jansen & Ruhlman, 2019). Over the past decade, next generation sequencing technologies have been used to generate hundreds of complete chloroplast genomes (Tangphatsornruang et al., 2010; Tangphatsornruang et al., 2011; Uthaipaisanwong et al., 2012). Recently, a number of complete chloroplast genomes of mangroves in the family Rhizophoraceae were reported (Li et al., 2019; Yang et al., 2019; Zhang, Zhong & Yuan, 2019; Shi et al., 2020; Li et al., 2021). These mangrove chloroplast genomes are 160–164 kb in length and consist of all four regions with LSC; ~91–93 kb, SSC; ~16–19 kb and IRs; ~26–27 kb. They contain 128–131 genes and have a highly conserved organization and structure. To date, only the chloroplast genomes of *B. sexangula* and *B. gymnorhiza* are available (Shi et al., 2020; Li et al., 2021). Thus, the chloroplast genome sequences in the genus *Bruguiera* are limited.

In this study, the chloroplast genomes of five *Bruguiera* species (*B. cylindrica, B. gymnorhiza*, *B. hainesii*, *B. parviflora* and *B. sexangula*) in the family Rhizophoraceae were sequenced, assembled and compared to reveal their evolutionary relationship. These chloroplast genomes will provide valuable genetic information and better understanding of evolutionary relationships among *Bruguiera* species.

## Materials & Methods

### DNA extraction and sequencing

Fresh leaves in five *Bruguiera* species (*B. cylindrica*, *B. gymnorhiza*, *B. hainesii, B. parviflora*, and *B. sexangula*) were collected from Ranong province in Thailand (Table S1) and stored in liquid nitrogen. DNA samples were extracted using a standard CTAB (Cetyl Trimethyl Ammonium Bromide) method (Doyle & Doyle, 1987). Then the genomic DNA was sequenced using an Illumina Hiseq2000 platform according to Illumina protocols.

### Chloroplast genome assembly and annotation

Based on a reference genome-based strategy, the Illumina paired-end reads and *B. sexangula* (the accession CNA0003536 in the project-ID CNP0000567 in CNSA (https://db.cngb.org/cnsa) (Shi et al., 2020)) were used for assembling the chloroplast genomes of the five *Bruguiera* species using GetOrganelle (Jin et al., 2020).

The chloroplast genome sequences were annotated using GeSeq (Tillich et al., 2017). The start-stop loci and intron-exon junctions of coding genes were checked manually based on reported chloroplast genes of other land-plant species. In addition to the coding genes, transfer RNAs (tRNAs) were identified using ARAGORN v1.2.36 (Laslett & Canback, 2004) in the GeSeq software. The circular structures of the chloroplast genomes were visualized using OGDRAW v1.3.1 (Greiner, Lehwark & Bock, 2019). The sequences of the five chloroplast genomes were deposited in GenBank (accession number MW836110–MW836114).

In addition, raw reads in each species were mapped to the *rpl32* sequence using BWA (Li & Durbin, 2009) and one primer pair was designed to amplify the *ndhF*-*rpl32* region (Table S2) (Shaw et al., 2007) in order to examine evidence for the *rpl32* gene loss in the assembled chloroplast-genome sequence of *B. gymnorhiza*.

### Comparative genome analysis

The comparison of whole chloroplast genomes among the *Bruguiera* species and two close non-mangrove species, *Pellacalyx yunnanensis* (MN106253) of Rhizophoraceae and *Erythroxylum novogranatense* (NC_0306061) of Erythroxylaceae, were analyzed to investigate sequence divergence using mVISTA with the Shuffe-LAGAN mode (Frazer et al., 2004). The previously reported chloroplast genome of *B. sexangula* was used as a reference for comparison (Shi et al., 2020). Furthermore, the junctions and borders of the IR regions were illustrated using IRscope (Amiryousefi, Hyvönen & Poczai, 2018).

### Repeat and SSR identification

Repeat sequences with length ≥50 bp were identified using REPuter (Kurtz et al., 2001). Furthermore, SSRs in the chloroplast genome sequences of the five *Bruguiera* species and three previously reported mangrove species, *Kandelia obavata* (MH277332), *Rhizophora x lamarckii* (NC_046517) and *Rhizophora stylosa* (MK070169), in the family Rhizophoraceae were identified using MISA (Thiel et al., 2003). Minimum repeat number requirements were set to 10 for mono-, five for di-, four for tri-, and three for tetra-, penta- and hexa-nucleotide repeats (Shi et al., 2020). The distance for compound SSRs was ≤100 bp (default).

Four polymorphic SSR loci were selected to examine polymorphism in different *Bruguiera* species. SSR primer pairs were designed for the four SSR loci and the resulting PCR product sizes were determined for each species (Table S3).

### Phylogenetic analysis

To validate the phylogenetic relationships of the *Bruguiera* species based on plastid genes, 9 representative families (Rhizophoraceae, Erythroxylaceae, Ctenolophonaceae, Violaceae, Salicaceae, Passifloraceae, Euphorbiaceae, Malpighiaceae and Clusiaceae) were selected from the order Malpighiales to construct phylogenetic tree. A total of 59 conserved chloroplast coding-genes in 40 species including the five species in this study, three other mangrove species, 31 land plant species and one outgroup species in the order Oxalidales were retrieved from NCBI and used to construct a phylogenetic tree (Table S4). These genes are *atpA*, *atpB*, *atpE*, *atpF*, *atpH*, *atpI*, *ccsA*, *cemA*, *clpP*, *matK*, *ndhA*, *ndhD*, *ndhE*, *ndhG*, *ndhH*, *ndhI*, *ndhJ*, *ndhK*, *petA*, *petD*, *petG*, *petL*, *petN*, *psaA*, *psaB*, *psaC*, *psaI*, *psaJ*, *psbA*, *psbC*, *psbD*, *psbF*, *psbH*, *psbJ*, *psbL*, *psbM*, *psbN*, *psbT, rbcL*, *rpl2*, *rpl14*, *rpl16*, *rpl33*, *rpl36*, *rpoA*, *rpoB*, *rpoC1*, *rpoC2*, *rps2*, *rps3*, *rps4*, *rps8*, *rps11*, *rps12*, *rps14*, *rps15*, *rps18*, *rps19* and *ycf3* (Table S4). Each coding-gene sequence was aligned using MUSCLE with default parameters in MEGA X (Kumar et al., 2018), and the aligned sequences in all genes were concatenated for each species. The best-fit model of plastid DNA substitution (GTR + I + G) was identified using the find best DNA/protein model tool in MEGA X. Based on the aligned sequences, a maximum likelihood (ML) phylogenetic tree was preformed using RAxML version 8.2.10 (Stamatakis, 2014) with GTRGAMMAI (GTR + I + G) model and 1,000 bootstrap values. *Averrhoa carambola* (NC_033350) in Oxalidales was used to be an outgroup species in the phylogenetic tree (Wurdack & Davis, 2009). The phylogenetic trees were visualized using FigTree v1.4.3 (http://tree.bio.ed.ac.uk/software/figtree/). In addition, chloroplast *rpl32* gene loss in *B. gymnorhiza* and other plant species in Malpighiales and Oxalidales (Alqahtani & Jansen, 2021) were plotted onto the their clades of the phylogenetic tree.

### Gene selective pressure analysis of *Bruguiera* chloroplasts

A total 74 shared plastid protein-coding genes in the five *Bruguiera* species and four terrestrial plant species, *Erythroxylum novogranatense* (NC_030601), *Ctenolophon englerianus* (NC_049158), *Averrhoa carambola* (NC_033350) and *Vitis rotundifolia* (NC_023790) (Table S5), were used to investigate selection pressures. Pairwise sequence alignments for each gene between the *Bruguiera* species and the terrestrial plant species based on the background of different evolutionary clades, including Erythroxylaceae (*E. novogranatense)*, Ctenolophonaceae (*C. englerianus*), Oxalidales (*A. carambola*), and Rosids (*Vitis rotundifolia*) (Han et al., 2020) were generated using MUSCLE with default setting in MEGA X (Edgar, 2004; Kumar et al., 2018). Gaps between compared sequences were removed before further analysis. Nonsynonymous (Ka) and synonymous (Ks) substitution rates (Ka/Ks) of each gene were calculated using KaKs Calculator version 2.0 (Wang et al., 2010), with its model-averaging method.

## Results

### Chloroplast genome features

**Figure 1:**
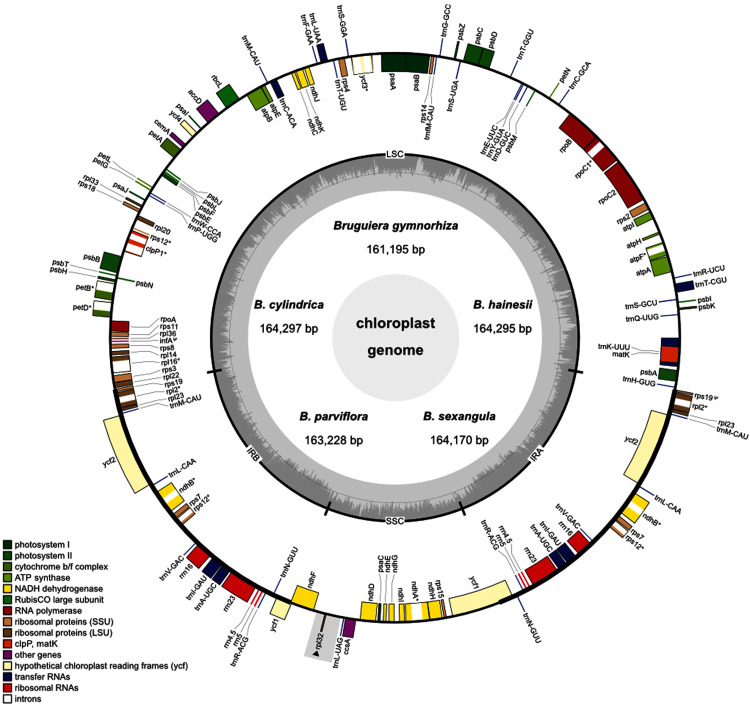
Circular map of the five *Brugueira* chloroplast genomes. The inner circle shows the GC content of the chloroplast genome. LSC, SSC, and IRs (IRA and IRB) are large single copy, small single copy, and inverted repeats, respectively. Genes based on different functional groups are shown in different colors. Grey rectangle indicates a loss region of *B. gymnorhiza*.

Five *Bruguiera* species (*B. gymnorhiza*, *B. hainesii, B. cylindrica*, *B. parviflora* and *B. sexangula*) were sequenced to generate 743,609,903–800,931,061 raw reads per species (150 bp in length) using Illumina Hiseq2000 data (Table 1). A total of 4,866,585–7,635,727 pair-end reads for each species mapped to the reference chloroplast genome of *B. sexangula* (Shi et al., 2020) and were used to assemble their chloroplast genome sequences. The read coverage was estimated at over 300× for each species. The size of the assembled chloroplast genomes ranged from 161,195 (*B. gymnorhiza*) to 164,297 (*B. cylindrica*) bp (Table 1; Fig. 1). Notably, the difference in genome length of *B. cylindrica* (164,297 bp) and *B. hainesii* (164,295 bp) was just 2 bp. All chloroplast genome sequences formed the typical quadripartite circular structure with the four regions including one large single copy (LSC), one small single copy (SSC) and two inverted repeat regions (IRA and IRB). The length of the LSC (91,154–91,738 bp) and IR (26,299–26,393 bp) regions was highly similar in all species, whereas the size of the SSC region had the highest variation and ranged from 17,053 (*B. gymnorhiza*) to 19,956 bp (*B. sexangula*) (Table 1). The average GC content in all chloroplast genomes was approximately 35%. The GC content in the IR regions (~42%) was higher than in the LSC (~33%) and SSC (~29%) regions.

**Table 1:** Summary of the chloroplast genome of five *Bruguiera* species.

Species	*B. gymnorhiza*	*B. cylindrica*	*B. hainesii*	*B. parviflora*	*B. sexangula*
Raw reads	743,609,903	791,571,475	755,597,881	781,228,900	800,931,061
Reads for chloroplast analysis	7,077,428	5,257,482	4,866,585	7,148,202	7,635,727
Base-coverage (x)	348	372	366	377	364
Genome size (bp)	161,195	164,297	164,295	163,228	164,170
LSC (bp)	91,404	91,738	91,736	91,154	91,428
SSC (bp)	17,053	19,839	19,839	19,476	19,956
IR (bp)	26,369	26,360	26,360	26,299	26,393
LSC GC content (%)	32.77	32.69	32.69	32.86	32.77
SSC GC content (%)	29.85	28.38	28.38	28.96	28.31
IR GC content (%)	42.19	42.20	42.20	42.26	42.19
Genome GC content (%)	35.54	35.22	35.22	35.43	35.25
No. of genes	129	130	130	130	130
No. of protein coding genes	82	83	83	83	83
No. of rRNAs	8	8	8	8	8
No. of tRNAs	37	37	37	37	37
No. of duplicated genes	17	17	17	17	17
Pseudogenes	2	2	2	2	2
Gene loss	*rpl32*	–	–	–	–

A total of 129 (*B. gymnorhiza*)–130 (*B. hainesii*, *B. cylindrica*, *B. parviflora* and *B. sexangula*) genes including 84–85 protein-coding genes, 37 transfer (tRNA) genes and eight ribosomal RNA (rRNA) genes were identified (Fig. 1, Tables 1 and 2). Of these, 19 genes (*ndhB*, *rpl2*, *rpl23*, *rps7*, *rps12*, *rps19*, *ycf1*, *ycf2*, *rrn4.5*, *rrn5*, *rrn16*, *rrn23*, *trnA-UGC*, *trnI-GAU*, *trnL-CAA*, *trnM-CAU*, *trnN-GUU*, *trnR-ACG*, and *trnV-GAC*) are duplicated in the IR regions (Fig. 1 and Table 2). All photosynthesis genes, including photosystems I (five genes) and II (15 genes), NADH dehydrogenase (11 genes), cytochrome b/f complex (six genes), ATP synthase (six genes) and rubisco large subunit (*rbcL*), were found in all *Bruguiera* chloroplast genomes. Several large and small subunits of ribosomal proteins (*rpl* and *rps*) were also identified (Table 2). Interestingly, a *rpl32* gene was lost only in *B. gymnorhiza* (Tables 1 and 2). The loss was detected from low read coverage in this region of *B. gymnorhiza* (Table S6) and confirmed by lack of a PCR product from the *ndhF*-*rpl32* region in *B. gymnorhiza* compared with other *Bruguiera* species (Fig. S1). Furthermore, other metabolic genes including maturase (*matK*), acetyl-CoA-carboxylate (*accD*), chloroplast envelope membrane protein (*cemA*), ATP-dependent protease (*clpP*), cytochrome c synthesis (*ccsA*) and conserved open reading frames (four genes) were found in all chloroplast genomes. There are two pseudogenes (*ycf1* and *rps19*) in the chloroplast genomes (Table 2). Among all identified genes, eight protein-coding genes (*atpF*, *ndhA*, *ndhB*, *petB*, *petD*, *rpl2*, *rpl16* and *rpoC1*) contain two exons and two protein-coding genes (*rps12* and *ycf3*) contain three exons (Tables 2 and S7). The introns in these genes in the *Bruguiera* species are highly conserved (Table S7). Several tRNA genes also contain introns such as *trnA-UGC*, *trnC-ACA*, *trnI-GAU*, *trnK-UUU*, *trnL-UAA* and *trnT-CGU*. The *trnK-UUU* intron in these chloroplast genomes encodes the protein-coding gene of *matK* (Fig. 1).

**Table 2:** List of annotated genes in the chloroplast genome of five *Bruguiera* species.

Category	Group of genes	Gene name
Photosynthesis	Subunits of Photosystem I	*psaA*, *B*, *C*, *I*, *J*
	Subunits of Photosystem II	*psbA*, *B*, *C*, *D*, *E*, *F*, *H*, *I*, *J*, *K*, *L*, *M*, *N*, *T*, *Z*
	Subunits of NADH dehydrogenes	ndhA*, B*(×2), *C*, *D*, *E*, *F*, *G*, *H*, *I*, *J*, *K*
	Cytochrom b6/f complex	*petA*, B*, D*, *G*, *L*, *N*
	ATP synthase	*atpA*, *B*, *E*, F*, *H*, *I*
	Rubisco	*rbcL*
Self-replication	Large subunit of ribosomal proteins	rpl2*(×2), *14*, 16*, *20*, *22*, *23*(×2), *32*^a^, *33*, *36*
	Small subunit of ribosomal proteins	*rps*2, *3*, *4*, *7*(×2), *8*, *11*, 12**(×2), *14*, *15*, *18*, *19*
	DNA dependent RNA polymerase	*rpoA, B*, C1*, *C2*
	rRNA genes	*rrn4.5*(×2), *5*(×2), *16*(×2), 23*(×2)
	tRNA genes	trnA-UGC*(×2), trnC-ACA*, *trnC-GCA*, *trnD-GUC*,
		*trnE-UUC*, *trnF-GAA*, *trnfM-CAU*, *trnG-GCC*,
		*trnH-GUG*, trnI-GAU*(×2), trnK-UUU*,
		*trnL-CAA*(×2), trnL-UAA*, *trnL-UAG*, *trnM-CAU*(×3),
		*trnN-GUU*(×2), *trnP-UGG*, *trnQ-UUG*,
		*trnR-ACG*(×2), *trnR-UCU*, *trnS-GCU*,
		trnS-GGA, trnS-UGA, trnT-CGU*, *trnT-GGU*
		*trnT-UGU*, *trnV-GAC*(×2), *trnW-CCA*, *trnY-GUA*
Other genes	Maturase	*matK*
	Subunit Acetyl-CoA-Carboxylate	*accD*
	Envelop membrane protein	*cemA*
	Proteaese	*clpP* **
	C-type cytochrome synthesis gene	*ccsA*
Unknows	Conserved Open reading frames	*ycf1*, *2*(×2), 3**, *4*
	pseudogene	*ycf1*, *rps19*

**Notes:**

* Gene with one intron.

** Gene with two introns.

a Loss gene in *B. gymnorhiza*.

### Chloroplast genome comparison

**Figure 2:**
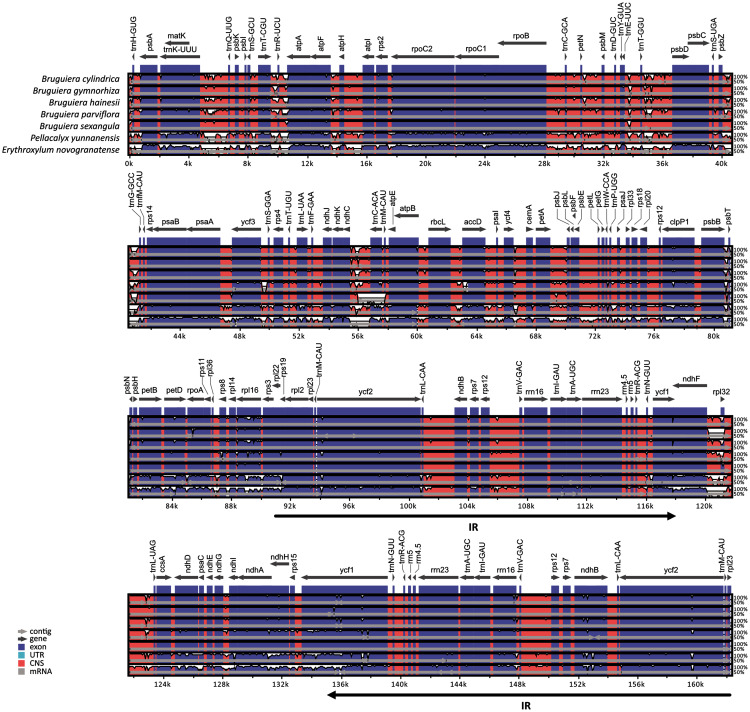
Alignment of the chloroplast genomes of five *Bruguiera* and two close non-mangrove species. The sequence identity was plotted among the chloroplast genomes, with *B. sexangula* (CNA0003536) as a reference using the mVista software. Grey and black arrows indicate genes and IR regions, respectively. Blue sections represent coding regions, and pink sections represent conserved non-coding sequences.

Comparison of chloroplast genomes in the five *Bruguiera* species and two closely related non-mangrove species (*P. yunnanensis* and *E. novogranatense*) revealed similar genome organization and gene order with some sequence variation (Fig. 2). Gene orientation was assessed by synteny analysis among the species, revealing conserved gene structure in the chloroplast genomes. The coding regions were more conserved than the intergenic regions. Both the coding and intergenic regions were highly similar in all *Bruguiera* species compared to the two non-mangrove species. The LSC and SSC regions had higher divergence than the IR regions indicating that the IR regions are highly conserved. A comparison between the two *B. sexangula* chloroplast genomes, previously reported (Shi et al., 2020) and the one generated in this study (Fig. 2), showed several small variations, including the difference of mononucleotide repeats, short repeats and indels. Furthermore, several poorly conserved regions located in the LSC (such as ~40.2–41.2 kb and ~55.5–56.8 kb) and SSC (such as ~120.0–121.3 kb) regions were found between the *Bruguiera* species and *E. novogranatense*. Some intergenic regions contained more variation among the species such as regions between *trnK*-*trnQ*, *trnT*-*trnR*, *trnR*-*atpA*, *trnT*-*psbD*, *psbZ*-*trnG*, *ndhC*-*trnC* and *ndhF*-*rpl32*.

**Figure 3:**
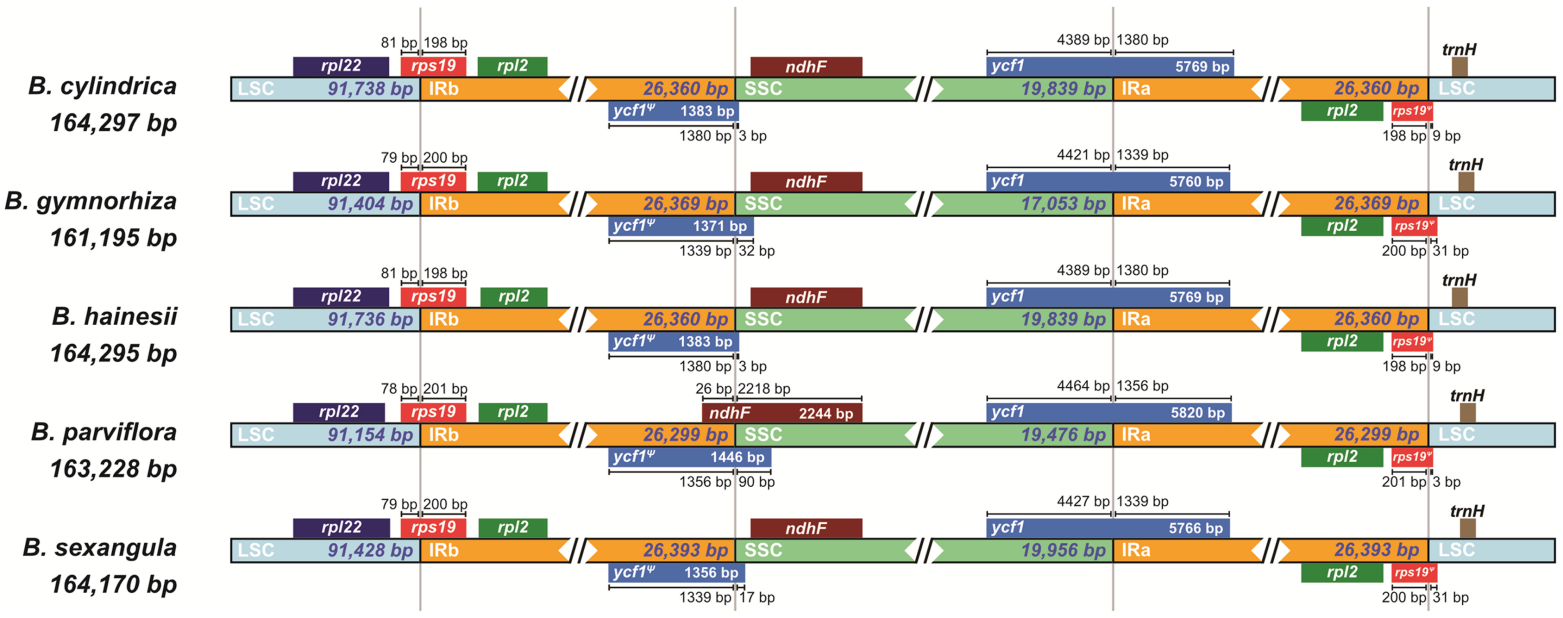
Comparisons of the borders of LSC, SSC, and IR regions among the five *Bruguiera* chloroplast genomes.

Moreover, the chloroplast boundary structures of the LSC, SSC and IRs were compared among the five species (Fig. 3). In all species, the *ycf1* gene and the *ycf1* pseudogene were located at the boundary of SSC/IRA and SSC/IRB, respectively, while the *rps19* gene and the *rps19* pseudogene were located at the boundary of LSC/IRB and LSC/IRA, respectively. The length of the *ycf1* and *rps19* pseudogenes varied from 1,371 bp (*B. gymnorhiza*) to 1,446 bp (*B. parviflora*) and from 204 bp (*B. parviflora*) to 231 bp (*B. gymnorhiza* and *B. sexangula*), respectively. The LSC/IR and SSC/IR borders of *B. cylindrica* and *B. hainesii* were more similar than the LSC/IR and SSC/IR borders of the other species. For example, the *ycf1* pseudogene is 3 bp away from the SSC/IRB border in both *B. cylindrica* and *B. hainesii*, whereas it is 32, 90 and 17 bp in *B. gymnorhiza*, *B. parviflora* and *B. sexangula*, respectively. The *rps19* pseudogene is 9 bp away from the LSC/IRA border in both *B. cylindrica* and *B. hainesii*, while it is 31, 3 and 31 bp in *B. gymnorhiza*, *B. parviflora* and *B. sexangula*, respectively. Additionally, the *ndhF* gene of *B. parviflora* straddles both the IRB and SSC boundary regions, with 26 bp of the IRB region and 2,218 bp of the SSC region, whereas the gene is located within the SSC region in all other species. While the sequence here is essentially the same in all species, *B. parviflora* has a variant that removes a stop codon causing *ycf1*^*ψ*^ to overlap *ndhF* in the SSC/IRB boundary region.

### Repeat and SSR contents

**Figure 4:**
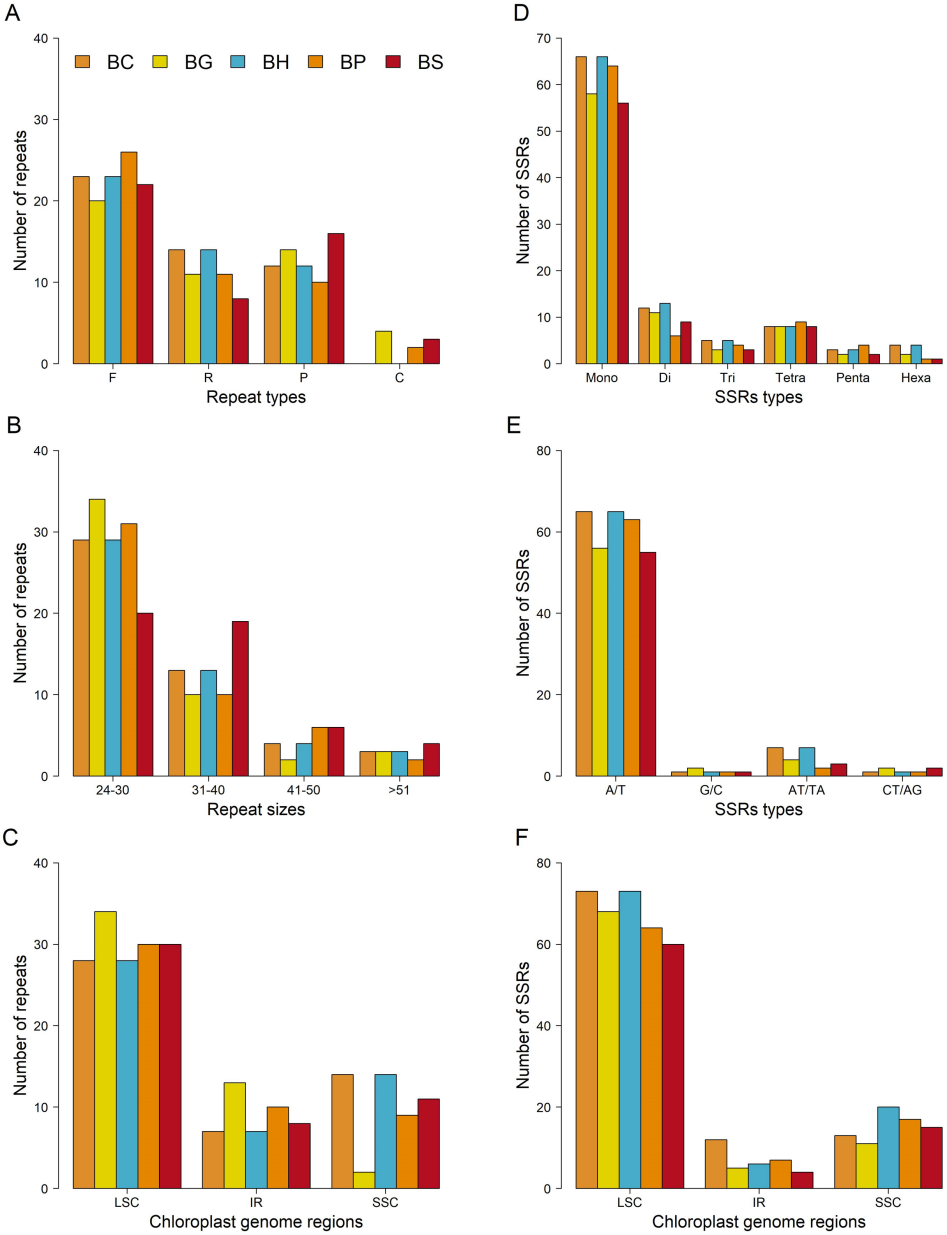
Comparison of repeats and microsatellites (SSRs) among the five *Bruguiera* chloroplast genomes. (A) Number of repeats based on types; (B) Number of repeats based on sizes; (C) Distribution of repeats in chloroplast regions; (D) Number of SSRs based on types; (E) Number of SSRs based on mononucleotides and dinucleotides; (F) Distribution of SSRs in chloroplast regions. BC, BG, BH, BP and BS represent *B. cylindrica*, *B. gymnorhiza*, *B. hainesii*; *B. parviflora* and *B. sexangula*, respectively.

A total of 49 repeats, consisting of forward, reverse, palindromic and complement repeats, were identified in the chloroplast genome of each *Bruguiera* species (Figs. 4A–4C and Table S8). Among them, the most common repeat type was forward repeat (Fig. 4A). The repeat patterns from *B. cylindrica* and *B. hainesii* were same. Most repeats ranged in length from 24 to 30 bp (Fig. 4B) and were in the LSC region (Fig. 4C).

SSR loci in the chloroplast genomes of the five *Bruguiera* species and three previously reported mangrove species in the family Rhizophoraceae were identified (Figs. 4D–4F; Tables 3 and S9). The number of SSR loci (simple, compound, and interrupted SSRs) varied among the species, ranging from 164 in *Kandelia obavata* to 197 in *B. hainesii* (Table 3). The number of SSR in *B. hainesii* (197) was higher than *B. cylindrica* (186) because of a variant that increased the repeat count of an SSR to reach the minimum threshold for inclusion. Among simple SSR, the number of mononucleotide repeats accounted for ~56–68% of all loci, of which A/T repeats were the most frequent type (Figs. 4D–4E). Most of the SSR loci were in the LSC region (Fig. 4F). In addition to simple SSR loci, there were numerous compound and interrupted SSR loci (more than two different repeat types present within ≤100 bases) (Tables 3, S9 and S10). Most *Bruguiera* species have more compound and interrupted SSR loci than other mangrove species in the family Rhizophoraceae. Each SSR loci were present in many *Bruguiera* species, with the most sharing between *B. cylindrica* and *B. hainesii*, and between *B. gymnorhiza* and *B. sexangula* (Table S10; Fig. S2). Two compound SSRs with 64 and 98 bp located in noncoding and intron-*ndhA* regions, respectively, were found in all *Bruguiera* chloroplast genomes. Notably, SSR loci of the *Bruguiera* chloroplast genomes were unique compared with the other mangrove species (*K. obavata*, *R. stylosa* and *R. x lamarckii*) in the family Rhizophoraceae.

**Table 3:** Number of SSRs in the chloroplast genomes of *Bruguiera* species and other reported mangrove species in the family Rhizophoraceae.

Species	SSR type						Total number	The number of SSRs for compound formation
	Mono-	Di-	Tri-	Tetra-	Penta-	Hexa-		
BH	119	31	16	16	9	6	197	61
BC	119	30	16	16	9	6	196	61
BG	102	26	14	18	4	2	166	51
BP	109	31	17	23	8	1	189	64
BS	109	36	16	19	5	1	186	70
BS^a^	106	31	14	18	5	1	175	58
KO	92	29	17	18	8	0	164	50
RL	117	22	13	10	9	2	173	52
RS	117	22	13	10	9	2	173	52

**Notes:**

BC, *B. cylindrica* (MW836110); BG, *B. gymnorhiza* (MW836111); BH, *B. hainesii* (MW836112); BP, *B. parviflora* (MW836113); BS, *B. sexangula* (MW836114); BS^a^, *B. sexangula* (CNA0003536); KO, *Kandelia obavata* (MH277332); RL, *Rhizophora x lamarckii* (NC_046517); RS, *Rhizophora stylosa* (MK070169) and PY, *Pellacalyx yunnanensis* (MN106253).

### Phylogenetic relationships

**Figure 5:**
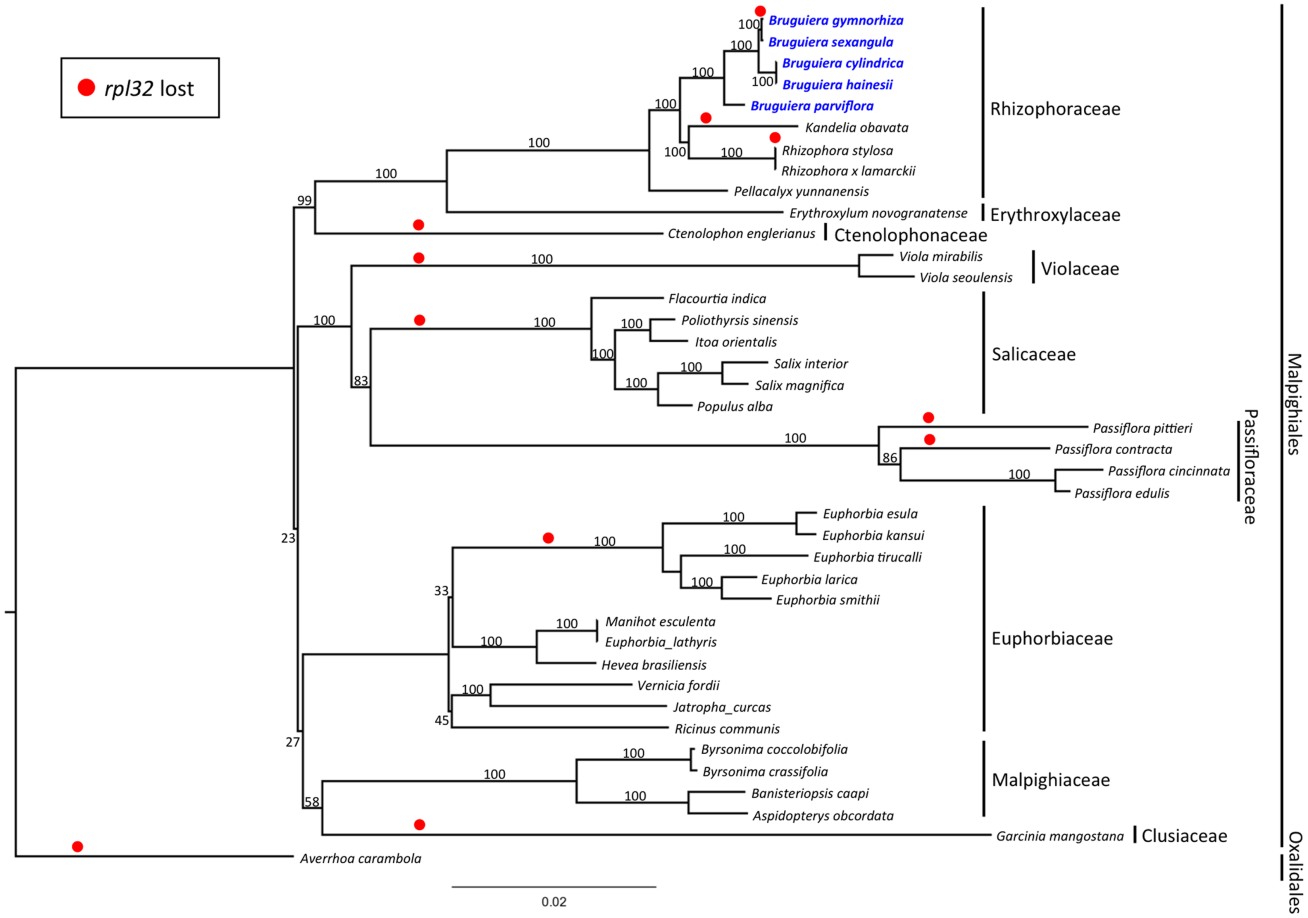
Molecular phylogenetic tree of the five *Bruguiera* species, three other mangrove species and thirty-two terrestrial plant species based on 59 conserved chloroplast coding genes. The maximum likelihood tree was constructed using RAxML program with the GTR + G + I nucleotide model. Numbers above nodes are bootstrap values with 1,000 replicates. *Averrhoa carambola* (NC_033350) was set as the outgroups. Loss of the *rpl32* gene is indicated by red circle.

Maximum likelihood (ML) phylogenetic analysis was performed based on 59 highly conserved chloroplast coding genes in 40 species including 39 plant species in Malpighiales and one outgroup species in Oxalidales (Fig. 5). The genetically distinct clades were classified into the lineages of nine families in Malpighiales consisting of nine Rhizophoraceae members, one Erythroxylaceae member, one Ctenolophonaceae member, two Violaceae members, six Salicaceae members, four Passifloraceae members, 11 Euphorbiaceae members, four Malpighiaceae members and one Clusiaceae member. The phylogenetic tree shows that all *Bruguiera* species were in the lineage Malpighiales and grouped under the clade Rhizophoraceae with a 100% bootstrap support. *B. gymnorhiza* was placed closely with *B. sexangula*, while *B. cylindrica* and *B. hainesii* were grouped together. *B. parviflora* is a sister species of the other four *Bruguiera* species. Loss of the *rpl32* gene was observed in several land-plant species (Fig. 5), such as *Ctenolophon engierianus*, *Viola* species, species in Salicaceae and *Euphorbia* species, and mangrove species such as *B. gymnorhiza*, *K. obavata* and *R. stylosa*.

### Selective pressure genes in *Bruguiera* mangrove species

To investigate gene selection pressures, the nonsynonymous and synonymous substitution (Ka/Ks) ratio was calculated by comparing 74 conserved chloroplast gene sequences of each *Bruguiera* species to assumed ancestors, which were *E. novogranatense* (Erythroxylaceae), *C. englerianus* (Ctenolophonaceae), *A. carambola* (Oxalidales) and *V. rotundifolia* (Rosids) (Table S11). Most Ka/Ks values of compared pairs were less than 1.0. There were two genes, *rps7* and *rpl36*, in which the Ka/Ks ratios were higher than one compared with *V. rotundifolia* (Rosids) and *E. novogranatense* (Erythroxylaceae), respectively suggesting positive selection during their evolution. The Ka/Ks ratios of the *rps7* gene between *B. cylindrica*, *B. gymnorhiza*, *B. hainesii*, *B. parviflora* and *B. sexangula* compared with *V. rotundifolia* were 1.58, 1.53, 1.58, 1.69 and 1.53, respectively. The Ka/Ks ratios of the *rpl36* gene between *B. cylindrica*, *B. gymnorhiza*, *B. hainesii*, *B. parviflora* and *B. sexangula* compared with *E. novogranatense* were 1.10, 1.10, 1.10, 0.94 and 1.10, respectively. The Ka/Ks ratios for the *rps7* gene were close to 1 when compared with *E. novogranatense* (Ka/Ks average of the five *Bruguiera* species = 0.91), *C. englerianus* (0.94), and *A. carambola* (0.97) (Table S11) revealing neutral selection within the lineages. The *rps7* gene of the *Bruguiera* species compared with *V. rotundifolia* contained 13 variation sites including 11 non-synonymous and two synonymous sites (Table 4). Five of the eleven non-synonymous sites (amino acid position: 21, 45, 105, 121, and 122) were unique to the *Bruguiera* species revealing specific nucleotide mutations during their evolution. Similar to the *rps7* gene, the *rpl36* gene of the *Bruguiera* species compared with *E. novogranatense* contained eight variation sites including six non-synonymous and two synonymous sites (Table 4). Three out of the six non-synonymous sites (amino acid position: five and 24 (two variation sites for one codon change)) were specific to the *Bruguiera* species.

**Table 4:** Variation sites in the *rps7* and *rpl36* genes between five *Bruguiera* species and four terrestrial plant species based on the reference sequence of *Vitis rotundifolia* and *Erythroxylum novogranatense*, respectively.

Gene	Nucleotide position	Nucleotide base									Amino acid position	Amino acid reference	Amino acid change
		*V. rotundifolia*	*A. carambola*	*C. emglerianus*	*E. novogranatense*	*B. cylindrica*	*B. gymnorhiza*	*B. hainesii*	*B. parviflora*	*B. sexangula*			
*rps7*	62	G	G	G	G	A	G	A	G	G	21	R	Q
	99	C	C	C	T	T	T	T	T	T	33	H	H
	133	C	C	C	C	G	G	G	G	G	45	R	G
	136	T	G	G	G	G	G	G	G	G	46	S	A
	139	G	A	A	A	A	A	A	A	A	47	V	M
	167	C	C	C	A	A	A	A	A	A	56	T	K
	173	C	C	C	G	G	G	G	T	G	58	P	R/L
	187	C	C	C	T	T	T	T	T	T	63	R	C
	314	C	C	C	C	T	T	T	T	T	105	S	F
	326	C	C	C	T	T	T	T	T	T	109	P	L
	363	A	A	A	A	T	T	T	T	T	121	L	F
	364	G	G	G	G	T	T	T	T	T	122	V	L
	372	T	T	T	C	C	C	C	C	C	124	A	A
*rpl36*	13	G	G	G	G	A	A	A	G	A	5	A	T
	19	G	G	A	A	G	G	G	G	G	7	I	V
	44	G	G	G	G	A	A	A	G	A	15	R	Q
	55	A	A	A	A	C	C	C	C	C	19	R	R
	63	A	A	A	A	A	A	A	G	A	21	G	G
	70	A	A	A	A	T	T	T	T	T	24	I	F
	72	A	A	A	A	C	C	C	C	C	24	I	F
	82	C	T	C	C	T	T	T	T	T	28	P	S

## Discussion

In the present study, we have determined the complete chloroplast genome sequence of five *Bruguiera* species using Illumina sequence data. The chloroplast genomes have highly conserved structure and gene content, as observed in most land plants (Daniell et al., 2016). The size of the chloroplast genomes ranged from 161–164 kb, concordant with other mangrove species (160–164 kb) in the family Rhizophoraceae (Li et al., 2019; Yang et al., 2019; Zhang, Zhong & Yuan, 2019; Shi et al., 2020; Li et al., 2021). The genomes consisted of four regions (LSC, SSC and two IRs) and the IR regions were highly conserved. Interestingly, *B. gymnorhiza* (161,195 bp), which is a widespread species in the Indo-West Pacific region, had a smaller chloroplast size than the other *Bruguiera* species (163,228–164,297 bp) because of a shorter SSC region compared with the others.

In all species, the chloroplast genomes contained the same number of tRNA (37) genes, rRNA (8) genes and pseudogenes (2), whereas the number of protein-coding genes (84–85) was slightly different. The *rpl32* gene in the SSC region was lost in *B. gymnorhiza*, which was validated by PCR amplification of the *ndhF*-*rpl32* region (Fig. S1). Loss of the *rpl32* gene was found in at least 73 species in eudicots, occurring through multiple independent loss events, such as *K. obovata* (mangrove) and *Populus* species (Ueda et al., 2007; Yang et al., 2019; Mohanta et al., 2020). The transfer of the *rpl32* gene from chloroplast to the nuclear genomes occurred several times across angiosperms (Ueda et al., 2007; Yang et al., 2019; Mohanta et al., 2020). In this study, we found that this gene was likely transferred to the nuclear genome of *B. gymnorhiza* due to low read coverage (~100×) compared with the other *Bruguiera* species (~8,000×) (Table S6). These results suggest that the *rpl32* gene was independently lost in chloroplasts of mangrove species.

The sequence and content of the IR regions were more conserved than those of the LSC and SSC regions in the *Bruguiera* species, consistent with other mangrove chloroplast genomes (Li et al., 2019; Yang et al., 2019; Zhang, Zhong & Yuan, 2019; Shi et al., 2020). The IR regions had high GC content, concordant with other mangrove plants (Li et al., 2019; Yang et al., 2019; Zhang, Zhong & Yuan, 2019; Shi et al., 2020). In addition, a contraction of the IR-SSC boundary region was found in *B. parviflora (*163,228 bp); as a result, its chloroplast genome size was smaller than those of *B. cylindrica*, *B. hainesii* and *B. sexangula* (164,170–164,297 bp). There was an overlap between *ycf1*^*Ψ*^ and *ndhF* in the SSC/IRB boundary region of *B. parviflora*, similar in several plant chloroplast genomes (Munyao et al., 2020; Su et al., 2020). These are common evolutionary events that result in size variation of chloroplast genomes in land plants (Wicke et al., 2011). Moreover, the genes adjacent to LSC/IR and SSC/IR boundaries are *rps19*, *rps19* pseudogene, *ycf1* and *ycf1* pseudogene with variation in pseudogene length, similar in many land plants (Gichira et al., 2019). In contrast, the boundary of LSC/IRA junction of *K. obovata*, which is a true mangrove species in the family Rhizophoraceae, was near the *rpl22* gene resulting in a shorter LSC and larger IR to give an overall larger chloroplast genome (Yang et al., 2019), revealing the variation of the IR junction among mangrove species during evolution.

A large number of repeats and SSRs were identified in the chloroplast genomes, that were mostly distributed in the LSC region as this contains a large amount of intergenic sequence. No differences were found between *B. cylindrica* and *B. hainesii* for the four SSR primer pairs (Fig. S2). *B. hainesii* was reported to be a hybrid between *B. gymnorhiza* and *B. cylindrica* identified using nuclear DNA markers (*CesA* and *UKN*) (Ono et al., 2016). The high similarity in chloroplast sequence and lack of genetic variation between *B. cylindrica* and *B. hainesii* are consistent with the chloroplast of *B. hainesii* originating from *B. cylindrica*. Comparison of SSRs, based on the same criteria of the SSR identification, in *B. sexangula* grown in two different locations (Thailand and China) revealed intraspecific sequence variation. For example, the Thai sample contained 186 SSRs, while only 175 SSRs were previously reported in the Chinese sample using the same criteria for SSR identification (Shi et al., 2020). The overall pattern of chloroplast SSR loci in the *Bruguiera* species were mostly unique compared with *Kandelia* and *Rhizophora* species. Thus, these SSRs would provide a useful genetic resource for future molecular marker analyses among *Bruguiera* species.

The ML phylogenetic tree was constructed based on 59 conserved chloroplast coding-genes in 40 species. The genus *Bruguiera* and other mangrove species, *K. obovata*, *R. stylosa* and *R. x lamarckii*, constituted a monophyletic branch of the clade Rhizophoraceae. Our phylogenetic result is consistent with morphological characters and previous phylogenetic results based on nuclear DNA markers and some chloroplast genes (*e.g*., *matK* and *rbcL*) (Schwarzbach & Ricklefs, 2000; Lakshmi, Parani & Parida, 2002; Sheue, Yong & Yang, 2005; Sahoo et al., 2007; Duke & Ge, 2011). For example, a phylogenetic tree was previously constructed using 34 land plant species including *Bruguiera* species (*B. cylindrica*, *B. exaristata*, *B. gymnorhiza*, *B. parviflora*, and *B. sexangula*) based on the *rbcL* gene and *trnL-trnF* spacer (Schwarzbach & Ricklefs, 2000). The phylogenetic result of these two sequences presented *B. gymnorhiza* and *B. sexangula* in the same clade. Another phylogenetic tree was reported based on chromosome and RAPD markers in four *Bruguiera* species that clustered into two groups with *B. gymnorhiza* and *B. sexangula* in one group, and *B. cylindrica* with *B. parviflora* in the other group (Sahoo et al., 2007). These results confirm the close relationships of *B. gymnorhiza* and *B. sexangula*.

The Ka/Ks values for all shared protein-coding genes of the *Bruguiera* species were calculated and compared with the four assumed ancestors to assess gene selection pressure. Among the protein-coding genes, nonsynonymous substitutions (Ka) were more frequent than synonymous substitutions (Ks) in *rps7* (11 non-synonymous sites of 13 variation sites) and *rpl36* (six non-synonymous sites of eight variation sites). The Ka/Ks values of the *rps7* and *rpl36* genes were above 1.0 in all species indicating that these genes were under positive selection. The result of the *rps7* gene is consistent with previous findings for many mangrove species and some land plants such as *K*. *obovata*, *R. stylosa*, *R. mangle* and *Ananas comosus* (pineapple) (Redwan, Saidin & Kumar, 2015; Han et al., 2020). The *rps7* gene encoding small ribosomal protein seven is involved in the regulation of chloroplast translation (Fargo, Boynton & Gillham, 2001). The *rpl36* gene was reported to be under positive selection in many mangrove species such as *Pemphis acidula*, and *Sonneratia ovata* (Shi et al., 2020). Knockout of the *rpl36* gene in tobacco results in severe morphological aberrations, poor photoautotrophic growth and low translational efficiency (Fleischmann et al., 2011). Therefore, the *rps7* and *rpl36* genes might be candidates for improving stress tolerance in mangrove species in dynamic and rapidly changing coastal environments.

## Conclusions

This study reports the whole chloroplast genome sequences of the five *Bruguiera* mangrove species. The comparative analyses among them provide insights into the chloroplast genome evolution in the genus *Bruguiera*. Evidence from the *Bruguiera* chloroplast genomes confirms that *B. hainesii*, a hybrid species between *B. cylindrica* and *B. gymnorhiza*, has inherited the chloroplast from *B. cylindrica*. Translocation of *rpl32* from the chloroplast to the nuclear genome was demonstrated to occur in only *B. gymnorhiza*; but not in other *Bruguiera* species. The comparison of the *Bruguiera* species in the family Rhizophoraceae based on phylogenetic relationships showed that *B. gymnorhiza* was closely related with *B. sexangula*, *B. hainesii* was confirmed to have inherited its chloroplast from *B. cylindrica*, and *B. parviflora* is a sister species of the other four *Bruguiera* species. Molecular markers were developed from SSRs and demonstrated to be useful in differentiating genotypes at both interspecific and intraspecific levels. The *rps7* and *rpl36* genes were found to be under positive selection in the *Bruguiera* species, and these genes might be important for stress tolerance. Therefore, these chloroplast genomes will provide useful genetic information for further analysis in *Bruguiera* and other mangrove species.

## Supplemental Information

10.7717/peerj.12268/supp-1 Supplemental Information 1 Sample location of five *Brugueira* species.

10.7717/peerj.12268/supp-2 Supplemental Information 2 Sequences of a primer pair for testing *rpl32* loss in five *Bruguiera* species.

10.7717/peerj.12268/supp-3 Supplemental Information 3 List of SSR primer pairs in five *Bruguiera* species.

10.7717/peerj.12268/supp-4 Supplemental Information 4 List of chloroplast accession numbers and genes for phylogenetic analysis.

10.7717/peerj.12268/supp-5 Supplemental Information 5 List of chloroplast accession numbers and genes for gene selective pressure analysis.

10.7717/peerj.12268/supp-6 Supplemental Information 6 The read coverage of the *rpl32* gene in five *Bruguiera* species.

10.7717/peerj.12268/supp-7 Supplemental Information 7 The chloroplast genes with exons and introns in five *Brugueira* species.

10.7717/peerj.12268/supp-8 Supplemental Information 8 Repeats of five *Bruguiera* species.

10.7717/peerj.12268/supp-9 Supplemental Information 9 SSRs of five *Bruguiera* species and three related mangrove species.

10.7717/peerj.12268/supp-10 Supplemental Information 10 Shared compound and interrupted SSR loci with ≤100 bp among five *Bruguiera* species.

10.7717/peerj.12268/supp-11 Supplemental Information 11 Ka/Ks values between *Bruguiera* species and assumed ancestors.

10.7717/peerj.12268/supp-12 Supplemental Information 12 Analysis of PCR products by 1% agarose gel electrophoresis. A gel purified PCR products of a primer pair, ndhF-F and rpl32-R (Table S2) in five *Bruguiera* chloroplasts. Lane M1 and M2: DNA size marker, Lane 1: PCR product in *Bruguiera cylindrica* (1,202 bp), Lane 2: PCR product in *B. gymnorhiza* (no product), Lane 3: PCR product in *B. hainesii* (1,202 bp), Lane 4: PCR product in *B. parviflora* (1,199 bp) and Lane 5: PCR product in *B. sexangula* (1,169 bp).

10.7717/peerj.12268/supp-13 Supplemental Information 13 Analysis of SSR markers. A set of 20 of SSR markers were used to estimate the genetic variation among five *Bruguiera* species. Y-axis is nucleotide size. A1–A5: SSR product of cpSSR1-F and cpSSR1-R primers (Table S3) in *Bruguiera cylindrica*, *B. gymnorhiza, B. hainesii*, *B. parviflora* and *B. sexangula*, respectively. A6–A10: SSR product of cpSSR2-F and cpSSR2-R primers in *B. cylindrica*, *B. gymnorhiza, B. hainesii*, *B. parviflora* and *B. sexangula*, respectively. A11–A15: SSR product of cpSSR3-F and cpSSR3-R primers in *B. cylindrica*, *B. gymnorhiza, B. hainesii*, *B. parviflora* and *B. sexangula*, respectively. A16–A20: SSR product of cpSSR4-F and cpSSR4-R primers in *B. cylindrica*, *B. gymnorhiza, B. hainesii*, *B. parviflora* and *B. sexangula*, respectively.

## Additional Information and Declarations

### Data Availability

The following information was supplied regarding data availability: All raw reads are available at Sequence Read Archive (SRA): SRR14318719 to SRR14318723. These Illumina reads were used to assemble the chloroplast genomes of five Bruguiera species. https://www.ncbi.nlm.nih.gov/Traces/study/?acc=PRJNA724818.
