# Supplementary material for: Complete chloroplast genome sequences of five Bruguiera species (Rhizophoraceae): comparative analysis and phylogenetic relationships
Source: PeerJ. 2021 Oct 22;9:e12268. doi: 10.7717/peerj.12268 (PMC8544253; doi:10.7717/peerj.12268)

Figure S1 Analysis of PCR products by 1% agarose gel electrophoresis.
A gel purified PCR products of a primer pair, ndhF-F and rpl32-R (Table S2) in five *Bruguiera* chloroplasts.
Lane M1 and M2: DNA size marker, Lane 1: PCR product in *Bruguiera cylindrica* (1202 bp),
Lane 2: PCR product in *B. gymnorhiza* (no product), Lane 3: PCR product in *B. hainesii* (1202 bp),
Lane 4: PCR product in *B. parviflora* (1199 bp) and Lane 5: PCR product in *B. sexangula* (1169 bp).

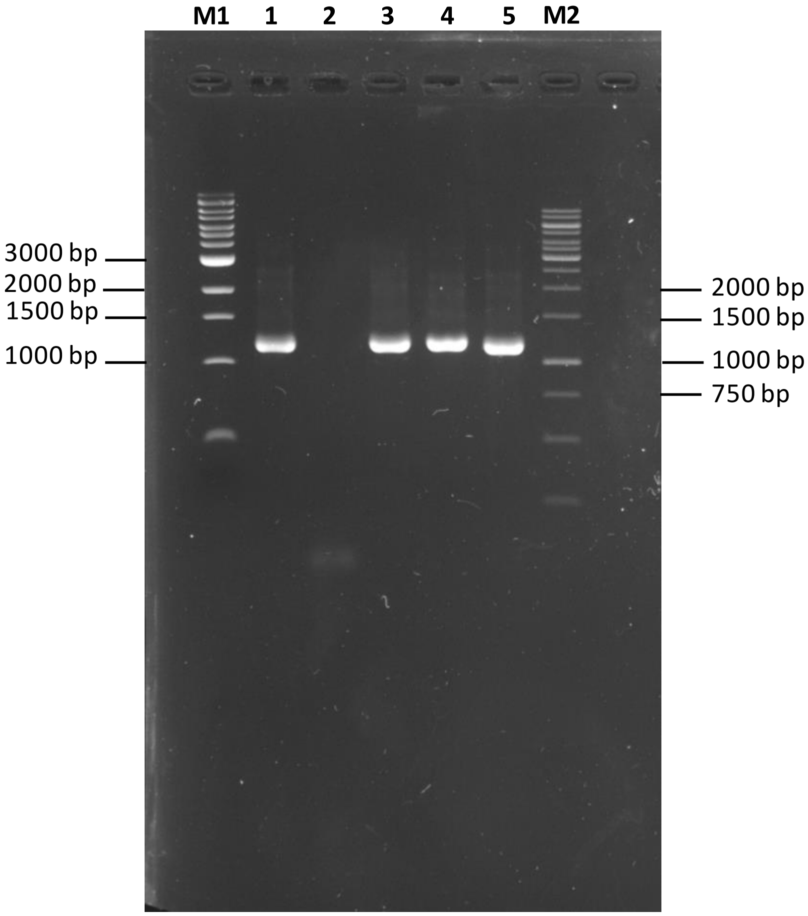

Supplement: Supplemental Information 12 — Lane M1 and M2: DNA size marker, Lane 1: PCR product in Bruguiera cylindrica (1,202 bp), Lane 2: PCR product in B. gymnorhiza (no product), Lane 3: PCR product in B. hainesii (1,202 bp), Lane 4: PCR product in B. parviflora (1,199 bp) and Lane 5: PCR product in B. sexangula (1,169 bp). [file peerj-09-12268-s012.pdf]
