# Supplementary material for: Complete chloroplast genome sequences of five Bruguiera species (Rhizophoraceae): comparative analysis and phylogenetic relationships
Source: PeerJ. 2021 Oct 22;9:e12268. doi: 10.7717/peerj.12268 (PMC8544253; doi:10.7717/peerj.12268)

Figure S2 Analysis of SSR markers.

A set of 20 of SSR markers were used to estimate the genetic variation among five *Bruguiera* species. Y-axis is nucleotide size. A1-A5: SSR product of cpSSR1-F and cpSSR1-R primers (Table S3) in *Bruguiera cylindrica*, *B. gymnorhiza, B. hainesii*, *B. parviflora* and *B. sexangula*, respectively. A6-A10: SSR product of cpSSR2-F and cpSSR2-R primers in *B. cylindrica*, *B. gymnorhiza, B. hainesii*, *B. parviflora* and *B. sexangula*, respectively. A11-A15: SSR product of cpSSR3-F and cpSSR3-R primers in *B. cylindrica*, *B. gymnorhiza, B. hainesii*, *B. parviflora* and *B. sexangula*, respectively. A16-A20: SSR product of cpSSR4-F and cpSSR4-R primers in *B. cylindrica*, *B. gymnorhiza, B. hainesii*, *B. parviflora* and *B. sexangula*, respectively.

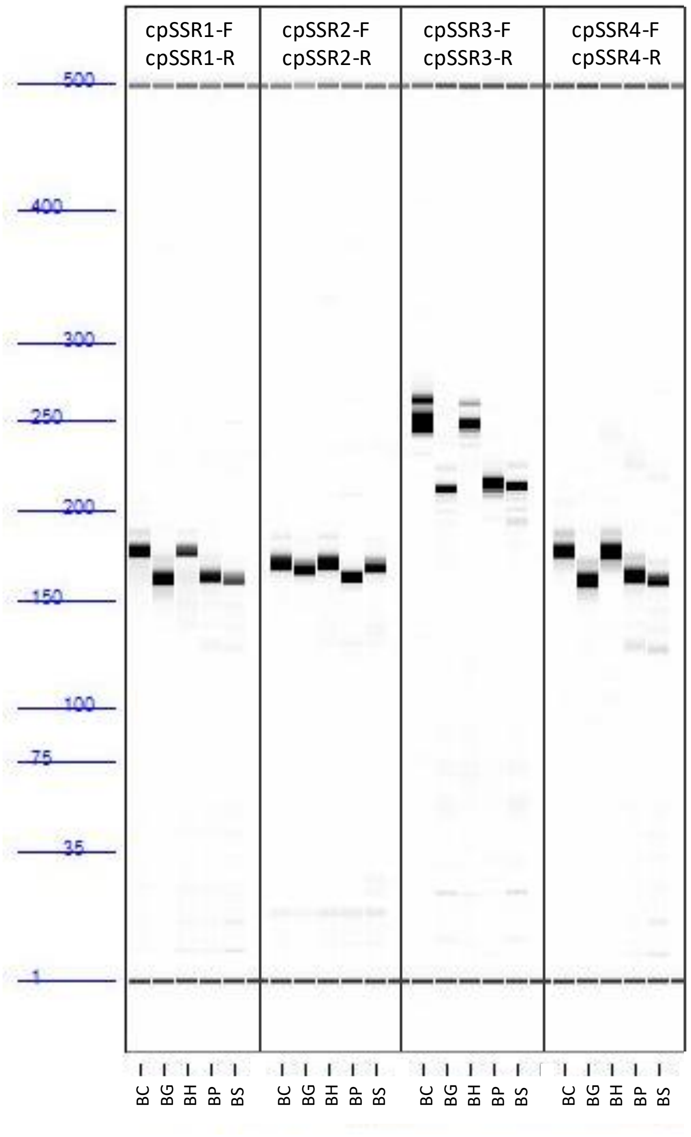

Supplement: Supplemental Information 13 — Y-axis is nucleotide size. A1–A5: SSR product of cpSSR1-F and cpSSR1-R primers (Table S3) in Bruguiera cylindrica, B. gymnorhiza, B. hainesii, B. parviflora and B. sexangula, respectively. A6–A10: SSR product of cpSSR2-F and cpSSR2-R primers in B. cylindrica, B. gymnorhiza, B. hainesii, B. parviflora and B. sexangula, respectively. A11–A15: SSR product of cpSSR3-F and cpSSR3-R primers in B. cylindrica, B. gymnorhiza, B. hainesii, B. parviflora and B. sexangula, respectively. A16–A20: SSR product of cpSSR4-F and cpSSR4-R primers in B. cylindrica, B. gymnorhiza, B. hainesii, B. parviflora and B. sexangula, respectively. [file peerj-09-12268-s013.pdf]
